# Intensity-modulated Radiation Therapy in the Management of Diffuse Choroidal Hemangioma in Sturge-Weber Syndrome

**DOI:** 10.18502/jovr.v20.15746

**Published:** 2025-07-10

**Authors:** Saeed Karimi, Sadra Ashrafi, Zahra Siavashpour, Mona Malekzadeh Moghani

**Affiliations:** ^1^Ophthalmic Research Center, Research Institute for Ophthalmology and Vision Science, Shahid Beheshti University of Medical Sciences, Tehran, Iran; ^2^Department of Ophthalmology, Torfeh Medical Center, Shahid Beheshti University of Medical Sciences, Tehran, Iran; ^3^Department of Radiation Oncology, Shohada-e Tajrish Hospital, Shahid Beheshti University of Medical Sciences, Tehran, Iran; ^4^Department of Radiotherapy, Shohada-e Tajrish Hospital, Shahid Beheshti University of Medical Sciences, Tehran, Iran; ^5^#First and second authors equally contributed to this work.

**Keywords:** Diffuse Choroidal Hemangioma, Intensity-modulated Radiotherapy, Sturge-Weber Syndrome

## Abstract

**Purpose:**

This study aimed to report the efficiency and safety of using intensity-modulated radiation therapy (IMRT) in treating diffuse choroidal hemangioma (DCH) in patients with Sturge-Weber syndrome (SWS).

**Methods:**

IMRT planning was carried out for each case after patient fixation, CT simulation, and target delineation. The purpose of treatment planning was to deliver the prescribed dose of 20 Gy to at least 95% of the planning target volume (PTV). The primary follow-up goal was to evaluate the efficacy and safety of IMRT as an alternative to traditional 3D conformal radiotherapy methods. The case series involved patients with DCH and varying degrees of vision impairment who underwent IMRT.

**Results:**

Five patients, comprising two men and three women, with an average age of 14.4 ± 3.78 years, were included in this study. These patients were followed up for an average duration of 14.4 ± 6.84 months. All patients exhibited notable reduction in subretinal fluid, significant tumor regression, and minimal side effects. Visual acuity improved in patients with pre-IMRT vision of hand motion or better.

**Conclusion:**

The findings suggest that IMRT is a promising, low-complication treatment option for managing DCH in SWS patients, warranting further research and potential integration into clinical practice.

##  INTRODUCTION

Sturge-Weber Syndrome (SWS) is recognized as a congenital neurocutaneous disorder with distinctive clinical features, including congenital port-wine facial nevi, glaucoma, seizures, and a range of neurological complications.^[1,2]^ A critical ocular manifestation of SWS is the development of choroidal hemangioma (CH).^[3,4]^ CHs are frequently diffused, on the same side as the skin lesions, and can lead to exudative retinal detachment (RD) and shifting subretinal fluid (SRF).^[5,6]^ The presence of such facial lesions and CHs is a significant medical concern due to their potential for visual impairment and pain; they can also pose substantial cosmetic issues that will seriously impact the quality of life of individuals with SWS.^[7]^

Management of CH in patients with SWS presents a significant clinical challenge. Traditional treatment methods, including laser therapy and systemic medications, have shown varying success rates in addressing this condition.^[8]^ Nevertheless, these conventional approaches are frequently associated with a spectrum of potential complications, including scarring, risk of tumor recurrence, and other systemic side effects. Moreover, there exists a subset of patients who exhibit suboptimal responses to these standard treatments, underscoring the necessity for exploring and evaluating alternative therapeutic strategies.^[1]^

External beam radiation therapy (EBRT) is a standard cancer treatment that involves the use of precisely focused radiation beams administered externally to eradicate internal cancerous tissues. This method has proved effective against diffuse choroidal hemangioma (DCH). However, the low conformity of basic 3D conformal radiotherapy (3DCRT) raises concerns about excessive radiation exposure to nearby healthy tissues and organs at risk (OARs).^[9]^ In such scenarios, intensity-modulated radiation therapy (IMRT) has emerged as a safer alternative, thanks to its capability to adjust and modulate beam intensity, thereby offering a more conformal, targeted, and less harmful treatment option.

IMRT represents a sophisticated advancement in radiation therapy and is characterized by the ability to precisely target tumor cells while preserving the integrity of the surrounding healthy tissues. This precision is particularly promising in treating DCH in patients with SWS.^[10]^ Initial studies and emerging evidence suggest that IMRT may offer substantial benefits in effective tumor management, relief from associated symptoms, and better cosmetic results.^[11,12]^ Despite these advantages, there remains a significant gap in research and patient-based reports that necessitates a thorough evaluation of the efficacy, safety, and long-term implications of IMRT in this distinctive patient group.

Considering the limitations in existing treatment modalities for DCH in patients with SWS, there is an urgent need to explore the application of IMRT for this condition. Patient reports following IMRT offer valuable information regarding treatment outcomes and possible complications. Therefore, this study aimed to determine the outcomes and side effects of IMRT in patients with DCH due to SWS. To the best of our knowledge, the present study is the first to investigate the efficacy and safety of IMRT in DCH management.

##  METHODS

### Study Setting and Population

This study was conducted as a prospective case series, approved by the Ethics Committee of the Ophthalmic Research Center, Research Institute for Ophthalmology and Vision Science at Shahid Beheshti University of Medical Sciences (IR.SBMU.ORC.REC.1402.021). Participants were carefully briefed about the treatment and potential side effects before their participation was confirmed through written consent. The study included patients ≥ 10 years old who had a confirmed diagnosis of DCH and massive exudative RD through clinical examinations and imaging. Patients who did not attend the scheduled follow-up visits were excluded from the study. Eligible patients were recruited from the retina clinic at Torfeh Hospital, Tehran, over a one-year period.

Upon enrollment, we collected demographic information, including age, gender, and medical history. We also performed a detailed ophthalmic evaluation, including a slit-lamp examination, fundoscopy, and assessment of the best-corrected visual acuity (BCVA) and intraocular pressure (IOP). Following the administration of IMRT as per our protocol, patients were scheduled for follow-up visits, initially every month for six months and subsequently every three months. During these follow-up sessions, patients received comprehensive eye examinations.

To assess treatment outcomes, we had to follow up with the patients for at least six months, and it was based on the results of the examination at the last follow-up session. The primary outcome measured in this study was tumor regression, which was determined by the reduction or elimination of massive SRF.

### Intensity-modulated Radiotherapy Protocol

Patient fixation was achieved using thermoplastic three-point head masks, an essential step for maintaining accurate positioning during treatment, especially for head and neck cases. The patients then underwent computed tomography (CT) with the fixation mask and a slice thickness of 1 mm. Magnetic resonance imaging (MRI) was also performed if needed, since it aids in target delineation for treatment planning. The image series was imported to the Eclipse treatment planning system (Version 13, Varian Medical Systems). Then, the CT and MRI of each case were fused. The radio-oncologist defined and contoured the gross tumor volume (GTV) and clinical tumor volume (CTV) on these images. Finally, to account for any potential internal target motion and setup errors, we added an additional margin of around 5 mm to the CTV to create the planning target volume (PTV) as the final target. We also delineated OARs, which were mainly eye globes, lenses, optic nerves, the optic chiasm, lacrimal glands, and brain stems. The treatment plan aimed to deliver the prescribed radiation dose of 20 Gy (in 10 fractions, i.e., 2 Gy per fraction) to at least 95% of the PTV.

**Figure 1:**
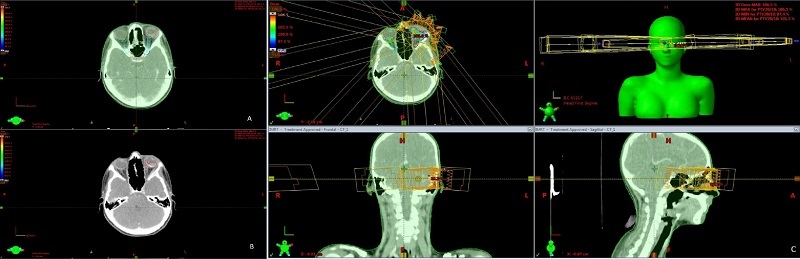
A sample of the IMRT treatment plan and dose distribution for one of the considered cases. (a) Planning tumor volume and organs at risk; (b) Clinical tumor volume; and (c) Fields and dose distribution of IMRT.

IMRT planning was performed by a radiotherapy medical physicist and evaluated and approved by the radio-oncologist while considering both planning objectives and the dose constraints of OARs. Eventually, all patients received IMRT using a 6 megavoltage photon beam of a Varian Linac (Clinac 600c, Varian Medical Systems) during 10 consecutive days, except weekends (Thursday & Friday) [Figure 1].

### Statistical Analysis

Demographic characteristics and treatment outcomes, specifically the resolution of SRF and tumor regression, were evaluated on a case-by-case basis. For the quantitative variables, the analysis involved calculating the mean and standard deviation (SD) for all patients.

##  RESULTS

In our study, five patients (two men and three women) with an average age of 14.4 ± 3.78 years were treated with IMRT for DCH and followed for an average of 14.4 ± 6.84 months. After treatment, BCVA for all patients either remained the same or improved; specifically, BCVA improved from hand motion (HM) to counting fingers (CF) in relevant cases, with no vision changes noted in patients with light perception (LP) or no light perception (NLP). No patient experienced increased IOP post-treatment, and those with previously elevated IOPs (cases 2 and 4) returned to normal levels during the follow-up period. The mean IOP decreased from 18.8 ± 7.42 mmHg before IMRT to 15.2 ± 3.11 mmHg at the final follow-up session. The observed radiotherapy-related complications were limited to a single case of cataract and one case of dry eye, with no further complications or tumor recurrence [Table 1]. The specifics of each patient's clinical progression post-treatment are detailed below.

**Table 1:** The demographic characteristics and clinical outcomes of IMRT in the cases

**Case**	**Age**	**Gender**	**Pre-IMRT VA**	**Final F/U VA**	**Pre-IMRT IOP**	**Final F/U IOP**	**SRF resolution**	**Radiation-related complications**	**F/U duration**
1	18	Male	HM	CF at 1 m	13	14	Complete	Dry eye	24
2	12	Female	NLP	NLP	28	18	Complete	Cataract	18
3	11	Female	HM	CF at 0.5 m	11	13	Complete	No	12
4	12	Female	LP	LP	25	12	Partial	No	6
5	19	Male	HM	CF at 1.5 m	17	19	Complete	No	12
HM, hand motion; F/U, follow-up; IMRT, intensity modulated radiation therapy; IOP, intraocular pressure; LP, light perception; NLP, no light perception; SRF, subretinal fluid; VA, visual acuity; CF, counting finger; m, meter									

**Figure 2:**
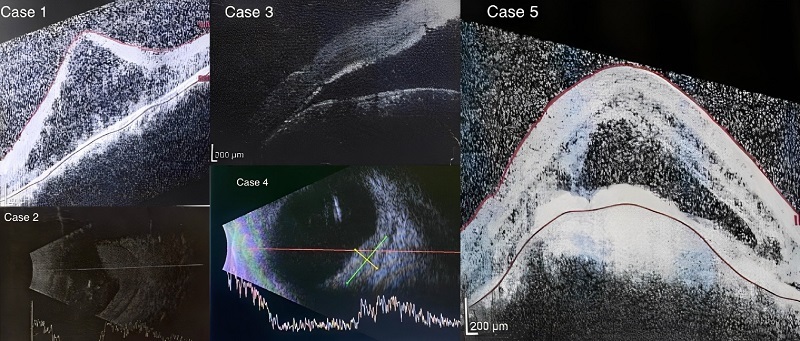
Subretinal fluid on OCT and B-scan of the five cases.

### Case 1

An 18-year-old male patient was admitted to the ophthalmology clinic with a primary concern of vision loss, which he associated with a minor head trauma several months earlier. He reported no history of systemic or ocular diseases. Clinical examination revealed a port-wine stain on the left half of his face. The BCVA in the left eye was noted as HM, and ocular movements were found to be normal. A slit-lamp examination disclosed the presence of a Shafer's sign in the anterior vitreous area. IOP was measured at 13 mmHg. During the fundoscopic examination, extensive exudative RD was observed. Given these findings, a provisional diagnosis of DCH was reached, and the patient was referred for optical coherence tomography (OCT), which confirmed the diagnosis. Subsequently, the patient underwent IMRT [Figure 2].

Follow-up visits at the retinal clinic were scheduled every two months. In the first post-IMRT visit, about a month later, the patient's only complaint was dry eyes, and the BCVA remained unchanged. IOP had increased slightly to 16 mmHg. Fundoscopic examination at this time showed a reduction in the RD, which was limited to the inferior area. By the fourth-month follow-up, vision improved to CF at 50 cm, and IOP was 18 mmHg. Furthermore, DCH regressed despite the persistence of SRF in the inferior area. Seven months post-treatment, SRF significantly decreased, and the patient's vision improved to CF at 1 meter. Fundoscopic examination revealed pigmentary lesions in the choroid, but DCH continued to regress.

Throughout the follow-up period, which extended to approximately two years post-treatment, the patient steadily improved. The BCVA in the left eye improved to CF at 1 meter compared to the pre-IMRT BCVA, and IOP stabilized at 14 mmHg. A mild posterior subcapsular (PSC) cataract was noted on slit-lamp examination. Fundoscopic examination at this stage showed an orange-colored subretinal lesion and pigmented chorioretinal areas, with no evidence of SRF. The OCT performed at this stage indicated a central macular thickness of 940 µm. The patient was advised to continue regular check-ups for monitoring his condition.

### Case 2

A 12-year-old female patient with a known diagnosis of SWS was referred to the retina clinic due to severely impaired vision in the right eye. The patient had no significant history of systemic or other ocular conditions. A notable port-wine stain was present on the right side of her face since birth. Examination revealed a positive relative afferent pupillary defect in the right eye, although eye movements were normal. The IOP in the right eye was elevated at 28 mmHg, leading to a diagnosis of neovascular glaucoma. The BCVA in this eye was recorded as NLP. Slit-lamp examination showed a mature nuclear sclerotic (NS) cataract in the right eye.

Due to advanced cataract, OCT was not feasible; thus, a B-scan ultrasound was performed. The ultrasound revealed extensive SRF, consistent with the diagnosis of DCH [Figure 2]. The patient underwent IMRT based on the established treatment protocol mentioned before. Concurrently, a regimen of topical glaucoma medications was initiated, including the combination of timolol and dorzolamide (Zilomole, Sina Darou Laboratories Company, Tehran) and latanoprost.

Two months post-IMRT, the patient's BCVA or slit-lamp examination remained unchanged. However, despite the regression of the hemangioma, the IOP increased to 38 mmHg, and the dosage of Zilomole eye drop increased in response. The follow-up B-scan indicated a significant reduction of SRF. At the five-month follow-up, the patient's vision remained unchanged, and the IOP was still uncontrolled at 37 mmHg. By this time, the DCH had undergone substantial regression.

During a visit 1.5 years after treatment, the patient's BCVA was unchanged (NLP). Slit-lamp examination revealed telangiectatic tortuous vessels, band keratopathy, and severe cataract. IOP was controlled at 18 mmHg. The B-scan performed at this stage confirmed complete resolution of SRF. Given the severe cataract and the high risk associated with phacoemulsification and posterior chamber intraocular lens (PE + PCIOL) surgery, the patient underwent corneal tattooing to mitigate the cosmetic effects of the cataract.

### Case 3

An 11-year-old female patient, previously diagnosed with SWS and without significant systemic illness, was presented to the eye clinic for retinal evaluation. Having a history of trabeculectomy, the patient reported decreased vision in the left eye to the level of HM. Initial assessment revealed a port-wine stain on the left side of the face. Ocular and pupillary movements were normal. Slit-lamp examination showed irregular, tortuous vessels in the conjunctiva. IOP was measured at 11 mmHg. Besides, extensive exudative RD was observed on fundoscopic examination.

After a diagnosis of DCH [Figure 2], the patient underwent IMRT according to the protocol established in previous cases. Two months post-treatment, the tumor regressed, and the SRF (predominantly localized in the inferior region) decreased significantly. The subsequent examinations over several follow-up visits showed no significant changes, and the patient reported no specific complaints.

At the last follow-up visit, approximately one year post-treatment, fundoscopic examination confirmed the regression of DCH, with no evidence of SRF. Notably, the patient's BCVA improved to CF at 50 cm. Additionally, the IOP was well-regulated at 13 mmHg. The patient was advised to continue with regular follow-up visits.

### Case 4

A 12-year-old female patient presented with a two-day history of pain and diminished vision in her right eye. Her medical history was notable for seizures, for which she was receiving treatment with valproic acid and lamotrigine. Clinical examination revealed a port-wine stain on the right side of her face. The BCVA in her right eye was limited to LP. Both pupil examination and eye movements were found to be normal. The IOP in the affected eye was elevated at 25 mmHg. Funduscopic examination identified a DCH accompanied by total exudative RD [Figure 2].

The patient was diagnosed with DCH and treated with IMRT according to the protocol outlined in previous cases. Concurrently, treatment with Zimolole eye drop was initiated due to elevated IOP. Given the patient's history of frequent seizures, a brain MRI was performed. The MRI findings confirmed reduced volume of the right brain hemisphere, leptomeningeal enhancement, and a decrease in T2 subcortical white matter signal.

At the six-month follow-up, the patient's BCVA remained unchanged, but IOP was normalized. Fundoscopic examination revealed a regression in the DCH and a reduction in SRF. The patient was advised to keep consistent follow-up appointments for ongoing monitoring of her status.

### Case 5

A 19-year-old male with a medical history of controlled IOP but no specific systemic diseases was admitted to the eye clinic due to progressive vision loss and discomfort in the left eye. Initial assessment of the left eye showed that BCVA was HM, with an IOP of 17 mmHg. Slit-lamp examination yielded normal results. However, funduscopic evaluation revealed SRF in the macula, and DCH diagnosis was established [Figure 2].

Following the diagnosis, the patient underwent IMRT according to the previously outlined protocol. At the one-month follow-up, there was no improvement in BCVA, although IOP decreased to 10 mmHg. Slit-lamp examination remained normal, and funduscopic assessment showed a reduction in SRF along with the presence of macular and peripapillary scarring.

Two months post-treatment, the patient's BCVA improved to CF at 1.5 meters, and IOP was slightly elevated to 11 mmHg. The slit-lamp examination revealed no new findings, and the funduscopic examination indicated a significant reduction in SRF and the regression of the DCH.

Three months later, no changes were reported on the patient's condition, and he expressed no additional concerns. At the last follow-up, one year after IMRT, the BCVA remained at CF at 1.5 meters with an IOP of 19 mmHg. The slit-lamp examination was normal, and the funduscopic examination confirmed the complete resolution of SRF and continued regression of DCH. Given the stability of the patient's condition, a follow-up visit was recommended in three months to prevent the emergence of new symptoms.

##  DISCUSSION

This study presents a case series of five patients with DCH associated with SWS who were treated with IMRT. The patients were of different ages and presented with varying degrees of vision impairment. In all cases, IMRT demonstrated efficacy in managing DCH. Significant regression of DCH was observed, which was associated with a reduction in SRF. IMRT was generally well-tolerated with minimal side effects, including dry eye in one case.

In the field of radiation oncology, IMRT emerges as a significant advancement, offering enhanced precision in targeting tumors. This technique provides a more accurate contouring of radiation doses around the tumor and minimizes exposure to adjacent normal tissues. The efficacy of IMRT in managing DCH is primarily attributed to these advantages. By improving the delineation of radiation dose profiles around tumors and at-risk nodal structures, this technique significantly reduces the overall toxicity profile associated with radiation therapy. A key objective of IMRT is to minimize the dose to normal tissue structures to preserve their function while ensuring full dose delivery to tumor targets. This is achieved through individually modulated fields or rotating linear accelerator gantry techniques, allowing for variable dose intensities through segments of each treatment field, thereby maximizing the conformality of the ultimate dose distribution.

The exploration of IMRT in clinical research has largely followed two basic approaches. The first approach focuses on maintaining the current rates of tumor control but with reduced toxic effects. In contrast, the second approach is more aggressive, aiming to increase the dosage to the tumor target, yet keeping toxicity within tolerable limits. This dual strategy highlights IMRT's versatility in balancing effective tumor management with patient safety.^[13]^

External beam radiotherapy (EBRT) has long been a treatment cornerstone for DCH in patients with SWS, showing promising results in SRF management in these patients. The shift toward IMRT represents a significant technological advancement, offering precise tumor targeting while minimizing radiation exposure to adjacent healthy tissues. Notably, we believe that none of the prior studies have explored the efficacy of IMRT in managing DCH, and they have instead focused on EBRT. This differentiation is crucial because the higher precision of IMRT significantly reduces the risk of radiation-induced side effects, a major limitation of traditional EBRT. In a retrospective study by Randon et al, which included 26 eyes from patients diagnosed with DCH, the patients were observed for a minimum duration of one year following EBRT. This study reported the recurrence of tumors in two patients, although no instances of eye enucleation were observed. Notably, six months post-radiation, a significant proportion of patients (65%) experienced a decline in BCVA. Additionally, two individuals developed glaucoma following EBRT. Among acute adverse effects, eyelid erythema, conjunctival hyperemia, and ocular discomfort were reported, whereas cataract formation was identified as a chronic complication in four patients. Contrary to these findings, no cases of tumor recurrence were noted in the current investigation, and radiation-induced glaucoma was absent in our patient cohort. The only side effect observed was dry eye syndrome, affecting a single patient in our study.^[14]^

Another study by Shilling et al evaluated the efficacy and safety of EBRT for managing circumscribed CH in 36 patients and DCH in 15 patients. Their findings indicated that 36% of patients with DCH retained SRF in regions distal to the fovea, necessitating additional interventions like laser photocoagulation. On the other hand, SRF was present in only one patient within our cohort. Moreover, Shilling et al documented vision loss in three patients following radiation therapy. In contrast, our analysis of patients treated with IMRT revealed no cases of vision impairment, underscoring the superior safety profile of IMRT over EBRT in treating DCH.^[15]^

In addition to EBRT, various older treatment modalities, such as laser photocoagulation and transpupillary thermotherapy, have been employed for managing DCH. However, these methods have become less attractive as primary treatments due to their lower efficacy and higher risk for complications. Laser photocoagulation was extensively used as the first choice for treating circumscribed CH, with effective fluid resolution in a significant percentage of cases, though often requiring multiple sessions and offering modest visual recovery. Transpupillary thermotherapy, a secondary option, has demonstrated success in fluid resolution and visual acuity maintenance only in selected cases of circumscribed CH.^[16,17]^

Photodynamic therapy (PDT) has also been widely adopted for circumscribed CH, showing tumor size reduction and SRF resolution. However, it is associated with minor complications like SRF recurrence, and its efficacy on DCH remains underexplored due to its lower energy beams and reliance on a photosensitizer.^[18,19,20]^ Conversely, IMRT, thanks to its higher energy beams, provides deeper penetration and higher efficacy for treating tumors, albeit with an increased risk of radiation-induced complications.

Brachytherapy, particularly with ruthenium-106, offers another effective CH treatment, improving vision and reducing tumor size, but also carries a risk of radiation complications.^[16,21,22]^ In contrast, IMRT offers significant advantages over brachytherapy. Its noninvasive nature eliminates the need for surgical procedures, reducing risks like infection and bleeding.^[23]^ IMRT's precision in targeting tumors minimizes radiation exposure to healthy tissues, leading to fewer complications such as tissue damage and cataracts.^[24]^ Thus, while brachytherapy is effective for CH treatment, IMRT presents a more advanced, safer alternative with greater precision and lower risk of side effects.

One limitation of this study is the small number of participants and the short follow-up period for assessing long-term complications. However, this study opens new avenues for further investigation. Future research should focus on the long-term effects of IMRT with a larger number of patients, its comparison with other treatments, and its impact on patients' overall quality of life. Moreover, due to the suboptimal quality of preoperative images, we could not confirm the observed clinical improvements in patients on the basis of comparative imaging evidence, which limits the ability to visually demonstrate IMRT's effectiveness. In future studies, it is crucial to ensure that high-quality images are captured both before and after treatment to more robustly validate clinical findings.

In summary, our research highlights the effectiveness of IMRT in treating DCH among patients with SWS. Our findings reveal that IMRT not only reduces tumor size but also involves a low rate of complications, making it a viable treatment option. However, the changes observed were solely based on clinical judgment and were not confirmed by any imaging modalities or measurements. The encouraging outcomes of our case series lay the groundwork for more extensive studies, potentially leading to enhanced treatment approaches in ophthalmic cancer care.

##  Financial Support and Sponsorship

None.

##  Conflicts of Interest

None.
